# Progress in Medicine

**Published:** 1896-09-26

**Authors:** 


### PROGRESS IN MEDICINE.

### RENAL DISEASES.

W. M. Ord[1] continues his interesting lectures on these diseases in the *Practitioner*. The hæmorrhages that occur with granular kidney are confessedly symptoms of great importance. Thus Ord would warn us against mistaking a bleeding from the lungs for a sign of pulmonary disease. Indeed, the cases of hæmoptysis, described by Sir Andrew Clark in elderly people, are related to the epistaxis and other hæmorrhages seen in granular kidney with high tension and diseased vessels. Whether we find albuminuria in these cases or not, they are a warning of possible cerebral hæmorrhage in the future.

Epistaxis may be a safety valve preventing the occurrence of hemiplegia, or worse. Dyspnœa may arise even in the prealbuminuric stage, sometimes with gastric disorders, either from temporary dilatation of the heart when the arterial tension is high, or from bronchial spasm. Later on true cardiac asthma becomes a terrible scourge, nitroglycerine with bromide and chloral are recommended as the best aid we can give (but refer below to Huchard's paper). The latter may be used hypodermically when there is vomiting. Ord does not fail to point out the danger of opium in these affections, and notices the curious fact that the distressing headache met with in some cases of high tension is due to cerebral anæmia, and that epileptic convulsions often follow a hæmorrhage or the application of leeches. Our aim must be to relieve the constriction of the vessels, which dams back the stream, not to cut off the force *a tergo*. Bonnier[2] suggests that professional cramp may be associated with Bright's disease, and gives notes of the case of a telegraph clerk, whose cramp came on after an attack of variola with nephritis, and who recovered when placed on a milk diet. Fresh investigations by Von Jaksch[3] show that uric acid exists in the blood in Bright's disease, especially in severe cases, and in uræmia. This confirms his previous experiments. Patients with high tension sometimes tolerate immense quantities of nitroglycerine. Thus, B. Robinson has given as much as a hundred grains in twenty-four hours without ill results.[4] At times it has no effect on the pulse, but in these cases chloral acts well, reducing the tension and giving sleep, as Ord notices.

**Uræmia.**—Amaurosis, sometimes of a transient character, may occur quite suddenly, and is usually referred to œdema or the action of toxins, but Friedel Pick[5] believes that actual areas of softening may be produced as a direct result of the uræmia. It is not however clear whether he would refer this to the effect of the toxins. Rendu,[6] too, discusses the production of uræmic aphasia, which he refers to uræmic poisoning. He treated a case successfully by bleeding and a daily injection of 40 centigrams of artificial serum. Word deafness is very rarely noted. A patient of Ballet's[7] showed marked but transient inability to understand words, though she could write and talk perfectly, and understand anything which was written. A slight degree of paraphasia lasting 24 hours occurred in the earlier stages, and two attacks of loss of consciousness. A large amount of albumen was found in the urine, and remained present there for ten days, and upwards, that is, during the period of observation. Though slight vertigo and headache were noted, there was no paralysis of the face or limbs. She was not at all deaf, but repeated to herself what was said, remarking that she could not understand. During the paraphasia she could name any object shown to her, but in talking used incorrect phrases and substituted one word for another. Another complication of uræmia is described by A. G. Barrs[8] as bullous dermatitis. The patient, a young woman, had suffered from œdema, albuminuria, dyspnœa, optic neuritis, and comatose states, and then developed an almost universal inflammation of the skin of a vesiculo-bullous type. The bullæ were especially large on the soles of the feet, and the eruption faded away under treatment before her death. A severe attack of albuminuria and uræmia with amaurosis occurred suddenly in a healthy girl under the care of Huchard[9] after the application of a cantharides blister to the epigastrium. Two other cases produced in the same way were mentioned by De Crésantigues, and Huchard believes that on account of this danger this form of blister should be entirely given up.

**Cancer.**—H. Armstrong[10] reports an instance of carcinoma affecting the left kidney, with a secondary growth blocking the right ureter. Complete anuria, with twitchings, amaurosis, and other symptoms, lasted for eight days, after which the patient appeared to be in good health. Another attack set in, and he died in coma. The recovery after eight days' anuria with such marked uræmic symptoms is unprecedented. Another case of cancer of the kidney is described by T. S. Short.[11] The points worthy of notice are (*a*) pain was felt for twelve months before any tumour could be felt by the hand; (*b*) growth afterwards was extremely rapid; (*c*) the seat of pain changed from time to time, but it was always worse on the right side, while the cancer was situated in the left kidney.

H. B. Allen and T. Cherry[12] contribute an interesting paper on the pathology of these tumours, and the question whether they should be classed as sarcomata or carcinomata. If they spring from the tubules, and if these tubules are epiblastic in origin, then we must regard them as carcinomatous. The authors examined a number of renal neoplasms, and came to the conclusion that so far as the new growths are concerned the renal epithelium behaves as a mesoblastic and not as an epiblastic formation, and that the tumours most closely resembling carcinoma in structure are not carcinoma in nature or history. The typical adenoma and sarcoma both pass insensibly at times into a pseudo-carcinomatous structure. Thus, they would not accept the theory that the epithelium of the straight tubes even is epiblastic, still less that of the convoluted ones. Moreover, all the tumours they examined appeared to spring from tubules, not from Wolfian or adrenal inclusions. Thus, in opposition to the common view, they appear to deny the existence of true carcinoma of the kidney. D. Newman,[13] in discussing the clinical symptoms of malignant growths, mentions that at first the lumbar pain is not increased by locomotion like that of calculas, and as time goes on it is paroxysmal, and extends to the lower extremities and elsewhere. The hæmorrhage is usually more profuse and less transient, and more often occurs at night when the patient is resting than in calculus. He upholds the distinction between the sarcomatous and carcinomatous growths, and shows that the extension of the latter takes place by the lymphatics.

[1] Practit., June. [2] Am. Jour. Med. Sciences, June. [3] B.M.J., July 18. [4] Med. Record, Ap. 11. [5] Internat. Med. Mag., March. [6] Med. Week, Ap. 3. [7] Med. Week, June 20. [8] Internat. Clinics, iv. 2. [9] Med. Week, March 27. [10] B. M. J., Ap. 18. [11] Birmingham Med. Rec., March. [12] Intercolonial Med. Jour., May 20. [13] Glasgow Med. Jour., March.

